# LMW Heparin Prevents Increased Kidney Expression of Proinflammatory Mediators in (NZBxNZW)F1 Mice

**DOI:** 10.1155/2013/791262

**Published:** 2013-09-17

**Authors:** Annica Hedberg, Premasany Kanapathippillai, Ole Petter Rekvig, Kristin Andreassen Fenton

**Affiliations:** RNA and Molecular Pathology Research Group, Institute of Medical Biology, Faculty of Health Sciences, University of Tromsø, 9037 Tromsø, Norway

## Abstract

We have previously demonstrated that continuous infusion of low molecular weight (LMW) heparin delays autoantibody production and development of lupus nephritis in (NZBxNZW)F1 (B/W) mice. In this study we investigated the effect of LMW heparin on renal cytokine and chemokine expression and on nucleosome-mediated activation of nucleosome-specific splenocytes. Total mRNA extracted from kidneys of heparin-treated or -untreated B/W mice was analysed by qPCR for the expression of several cytokines, chemokines, and Toll-like receptors. Splenocytes taken from B/W mice were stimulated with nucleosomes with or without the presence of heparin. Splenocyte cell proliferation as thymidine incorporation and the expression of costimulatory molecules and cell activation markers were measured. Heparin treatment of B/W mice reduced the *in vivo* expression of CCR2, IL1**β**, and TLR7 compared to untreated B/W mice. Nucleosome-induced cell proliferation of splenocytes was not influenced by heparin. The expression of CD80, CD86, CD69, CD25, CTLA-4, and TLR 2, 7, 8, and 9 was upregulated upon stimulation by nucleosomes, irrespective of whether heparin was added to the cell culture or not. In conclusion, treatment with heparin lowers the kidney expression of proinflammatory mediators in B/W mice but does not affect nucleosomal activation of splenocytes.

## 1. Introduction 

Systemic lupus erythematosus (SLE) is a chronic autoimmune syndrome characterized by inflammation and damage in several organs [1]. Lupus nephritis is one of the most severe manifestations of SLE, and autoantibodies against nuclear components such as dsDNA and nucleosomes are central in the development of the organ disease. These autoantibodies are found together with chromatin in electron dense structures (EDS) located in the mesangial matrix (MM) and glomerular basement membranes (GBM) of nephritic kidneys, as demonstrated in both murine [2–4] and human [5] forms of lupus nephritis. Several studies have demonstrated that the main autoantigen in lupus nephritis, assumingly serving as both inducer and target for the immune system, indeed is chromatin fragments or nucleosomes [6–9].

Studies have shown that SLE patients and lupus prone mice are assumed to suffer from impaired clearance of apoptotic debris [10, 11]. This may result in an increased load of extracellular chromatin and formation of immune complexes (ICs) [12–14]. Deposition of ICs within the MM and GBM is associated with renal expression of proinflammatory chemokines attracting leukocytes in SLE patients and murine models of lupus-like nephritis [15–17]. This will lead to increased influx of Fc receptor bearing effector cells activated by circulating ICs, which together will increase the ongoing inflammation and tissue destruction [18]. Interfering with activation of intrinsic kidney cells and effector cells may prevent or lower the expression of cytokines and chemokines.

We have previously demonstrated that lupus prone (NZBxNZW)F1 (B/W) mice receiving low molecular weight (LMW) heparin showed delayed anti-dsDNA antibody production compared to sham-treated control mice [19]. *In vitro* studies also showed that LMW heparin inhibited chromatin binding to components of GBM, and heparin increased enzymatic degradation of chromatin, as demonstrated using Dnase1 and proteinase K enzymes [19]. The aim of this study was to investigate if LMW heparin treatment, by preventing binding of ICs to the GBM, had an effect on cytokine, chemokine and Toll-like receptor mRNA expression profiles during the development of lupus nephritis and if heparin could prevent nucleosomal activation of splenocytes.

## 2. Materials and Methods

### 2.1. Ethics Statement

The treatment and care of animals were conducted in accordance with the Norwegian Animal Experimental and Scientific Purposes Act of 1986. All experimental protocols were approved by the Norwegian Animal Research Authority (NARA).

### 2.2. Mice and Grouping of Mice

Female B/W and BALB/c mice were purchased from Jackson Laboratory (Bar Harbor, Maine, USA). The B/W mice were divided into 4 groups based on age, deposition of IgG in glomeruli, anti-dsDNA ab titers in sera taken at end point, proteinuria, and heparin treatment. Group 1 (4–10 w.o, *n* = 17) had no depositions of IgG within the kidneys and no detectable levels of anti-dsDNA antibodies in sera. Deposition of IgG was observed within the kidney of Group 2 (mesangial nephritis determined by mesangial deposits, 18–30 w.o, *n* = 15) and Group 3 (end-stage organ disease determined by GBM deposits and proteinuria, 23–36 w.o, proteinuric, *n* = 18) B/W mice with detectable levels of anti-dsDNA ab in sera. In the heparin-treated group (Group 4, *n* = 5) 1/5 mice were anti-dsDNA antibody negative, and 2/5 mice developed proteinuria [19]. Age-matched BALB/c mice was used as controls.

### 2.3. Isolation of Kidneys from B/W Mice

B/W mice at age 4 weeks old (w.o) until the development of severe proteinuria (23–40 w.o) and age-matched control BALB/C mice were sacrificed in groups of 3 as described previously [4]. The heparin-treated mice included five mice given a daily subcutaneous dose of 50 *μ*g of Klexane (LMW heparin, Aventis Pharma AS) by osmotic pumps (Scanbur, Oslo, Norway) from the age of 12 weeks [19]. The osmotic pumps were primed and filled according to the manufacturer's instruction, implanted subcutaneously in the upper dorsal region, and replaced every ~30 days. Control mice received saline by saline-filled osmotic pumps. The heparin-treated mice were paired randomly with saline-treated control mice. The pairs of BW mice were sacrificed when the control mice developed full-blown lupus nephritis (31–39 w.o). The kidneys were isolated and processed for RNA isolation, immunohistochemistry (IHC) analysis, and immune electron microscopy (IEM) analysis, as described in [4].

### 2.4. Determination of Proteinuria and of Anti-dsDNA Antibodies by ELISA

Full-blown lupus nephritis was defined when proteinuria reached 4+, as determined by urine stix (Bayer Diagnostics, Bridgend, UK): 0-1+ (<1 g protein/liter urine) was regarded as physiological proteinuria; 2+ (≥1 g/liter to <3 g/liter) was regarded as mild proteinuria, and 3+ (≥3 g/liter to <20 g/liter) and 4+ (≥20 g/liter) was regarded as heavy proteinuria. Sera were collected and stored at −20°C until use. Serum antibodies against dsDNA were detected by ELISA as described in [20, 21]. Sera were diluted twofold from 1/100 to 1/6400 in PBS (0.02% Tween), and the 163c3 anti-dsDNA mAb (provided by T. N Marion, Memphis, TN, USA [22]) was included in each ELISA for assay validation and determination of cut-off value. 

### 2.5. Immunohistochemistry

Detection of autoantibodies bound in glomeruli was performed on Zink-fixed kidneys embedded in paraffin. Four *μ*m sections of the kidney samples were dewaxed with xylene, rehydrated in graded series of ethanol before blocking with 3% H_2_O_2_ to neutralize endogenous peroxidase. Sections were further blocked with 10% goat serum and 1% BSA in PBS before incubation with anti-mouse IgG antibodies conjugated with horseradish peroxidase (Sigma-Aldrich, St. Louis, MO, USA) diluted 1 : 100 in blocking solution. Washed sections were then incubated with chromogen DAB (Dako, Glostrup, Denmark) for detection of primary antibody. IHC using Polink-2 Plus HRP detection kits for tissue (Golden Bridge International, Inc, Mukilteo, WA, USA) was performed on frozen kidney sections. Antibodies against mice CCR2 and TLR7 were purchased from Abcam (Cambridge, UK), and IL1*β* was obtained from R&D systems (Minneapolis, USA).

### 2.6. Isolation of Splenocytes from B/W Mice

Spleens were collected and mashed through a 100 *μ*m cell strainer with DMEM-10 (4.5 g/L glucose, 10% fetal bovine serum, 10000 U/mL penicillin, and 10 mg/mL streptomycin and L-glutamine) (Invitrogen, Carlsbad, CA, USA). Erythrocytes were lysed with ACK lysis buffer (150 mM NH_4_Cl, 10 mM KHCO_3_, and 0.1 mM Na_2_EDTA adjusted to pH 7.2–7.4), and splenocytes were washed and resuspended in DMEM-10.

### 2.7. Proliferation Assay

Splenocytes, at 10^5^ cells/200 *μ*L/well, were seeded out in a 96-well round bottom plate. The cells were incubated with different stimulators (all in triplicates): nucleosomes (10 *μ*g/mL) prepared from the murine BALB/c 3T3 clone A31 fibroblast cell line (ATCC CCL-163) and characterized as previously described in [23], nucleosomes (10 *μ*g/mL) together with LMW heparin (Enoxaparin, Klexane, Aventis Pharma AS, Oslo, Norway, 24 *μ*g/mL) at a molar ratio of 1 : 100 (nucleosomes (determined as core nucleosome equivalents): heparin), LMW heparin (24 *μ*g/mL), concanavalin A (con A) (2.5 *μ*g/mL) (Sigma-Aldrich), and conA with LMW heparin (1 : 200) and HMGB1 (1 *μ*g/mL) (Sigma-Aldrich). Tritiated thymidine (1 *μ*Ci/well) (Perkin Elmer, MA, USA) was added to the cell cultures 16 hours before harvesting at 20 h, 4 days, and 7 days. Cells were transferred to filter paper, each spot representing one well was isolated, and three mL of scintillation fluid (Ultima gold XR, Perkin Elmer) was added to each piece of filter paper. Counts per minute (cpm) were measured using a liquid scintillation analyzer (1900 TR, Packard Instruments). A proliferative response was defined as a stimulation index (SI) calculated as the mean cpm value for stimulated cells in triplicates divided with mean cpm value for medium stimulated cells at the same time point. Positive proliferation was regarded if SI was greater than 2, provided that the cpm of antigen-stimulated cells was above 100 cpm. 

### 2.8. Western Blot

Western blot was performed with SDS-NuPage-gels and blotting system according to the manufacturer (Invitrogen). Rabbit anti-mouse HMGB1 antibody (Sigma-Aldrich) was used for detection of HMGB1. Recombinant HMGB1 protein (Sigma-Aldrich) was used as control (31 kDa on SDS-PAGE). 

### 2.9. RNA Isolation

Differently stimulated splenocytes (all in triplicate) were harvested for total RNA isolation at the same time points as for the proliferation assay. Cells were collected and washed in ice cold PBS before isolating RNA with Trizol reagent (200 *μ*L) (Invitrogen) according to the manufacturer's protocol with minor modification. Chloroform (40 *μ*L) was used for phase separation, and RNA was precipitated with 96% ethanol containing 0.3 M NaAc and 20 *μ*g/mL glycogen. RNA was washed in 80% ethanol and dried before dissolving in Rnase-free water. The concentration and quality of extracted RNA were determined spectrophotometrically using NanoDrop (NanoDrop technologies, Wilmington, USA).

### 2.10. Gene Expression Analysis

Preparation of cDNA and quantitative PCR (qPCR) was performed exactly as described in [24]. The following TaqMan gene expression assays were used: Mm00446973_m1 for TBP as housekeeping gene, Mm01183378_m1 for CD69, Mm01340213_m1 for CD25, Mm00434256_m1 for IL2, Mm00515420_m1 for CD19, Mm00486849_m1 for CTLA-4, Mm01157262_m1 for TLR8, Mm00711659_m1 for CD80, Mm00444543_m1 for CD86, Mm00446590_m1 for TLR7, Mm00446193_m1 TLR9, Mm01210732_g1 for IL6, Mm00433859_m1 for CXCL1 (KC), Mm99999062_m1 for IL10, Mm99999061_mH for IL1*β*, Mm01168134_m1 for IFN-*γ*, Mm00443258_m1 for TNF*α*, Mn00441242_m1 for CCL2, Mn00438270_m1 for CCR2, Mn01308393_g1 for CCL7, Mn00444228_m1 for CCL20, Mn00436450_m1 for CXCL2, Mn00436451_g1 for CXCL5, and Mn00442346_m1 for TLR2. TaqMan Fast Universal PCR master mix (2X) and gene expression assays were all obtained from Applied Biosystems. Medium stimulated cells at each time point (20 hours, 4 days, and 7 days) served as reference, and changes in gene expression were calculated with the ΔΔCT method shown as fold change. 

### 2.11. Measurements of Cytokines in Cell Supernatants

Cytokine analyses were performed with ELISA MAX Standard Sets for mouse IL10 (BioLegend, San Diego, CA, USA) or mouse TNF*α* ELISA kit (Thermo scientific, Rockford, IL, USA). All assays were performed according to the manufacturer's instructions. 

### 2.12. Statistical Analysis

Unpaired *t*-test was used to compare mean of two sets of measurements. Statistical comparisons of groups were made by one-way ANOVA followed by Bonferroni posttest. Statistical comparisons of treatment were analyzed using two-way ANOVA followed by Bonferroni posttest. All tests were performed using GraphPad Prism version 5.0.

## 3. Results

### 3.1. The Effect of LMW Heparin on *In Vivo* mRNA Expression Levels of Cytokines, Chemokines, Chemokine Receptor, and TLRs

**Figure 1:**
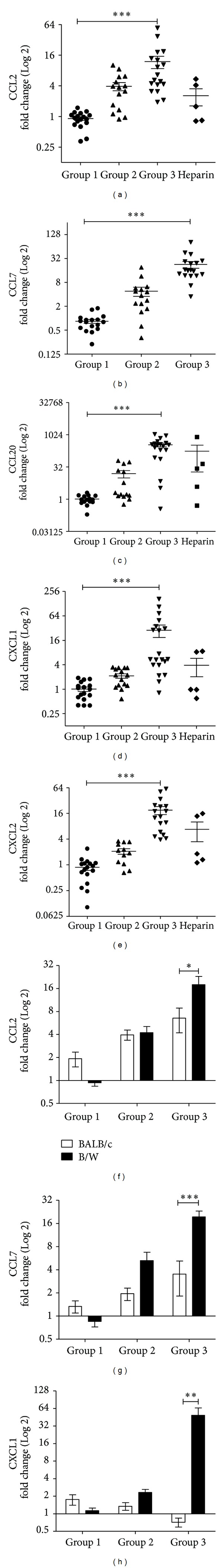
LMW heparin treatment does not affect chemokine mRNA expression within the kidneys of B/W mice. The mRNA expression of CCL2 (a), CCL7 (b), CCL20 (c), CXCL1 (d), and CXCL2 (e) was significantly increased in Group 3 mice and were not significantly reduced in heparin-treated mice. The mRNA expression of CCL2 (f), CCL7 (g), and CXCL1 (h) was also analyzed in age-matched BALB/c mice. Data is given as Log 2 of mean ± SEM of fold change values normalized against 4-week-old mice (*n* = 3). *P* values are calculated using one-way ANOVA followed by Bonferroni posttest. *P < 0.05; **P < 0.01; ***P < 0.001. Group 1: 4–10 w.o B/W mice (*n* = 17); Group 2: 18–30 w.o B/W mice with mesangial IC deposits without proteinuria (*n* = 15); Group 3: 23–36 w.o B/W mice with proteinuria (*n* = 18); Group 4: 31–39 w.o heparin-treated B/W mice (*n* = 5).

**Figure 2:**
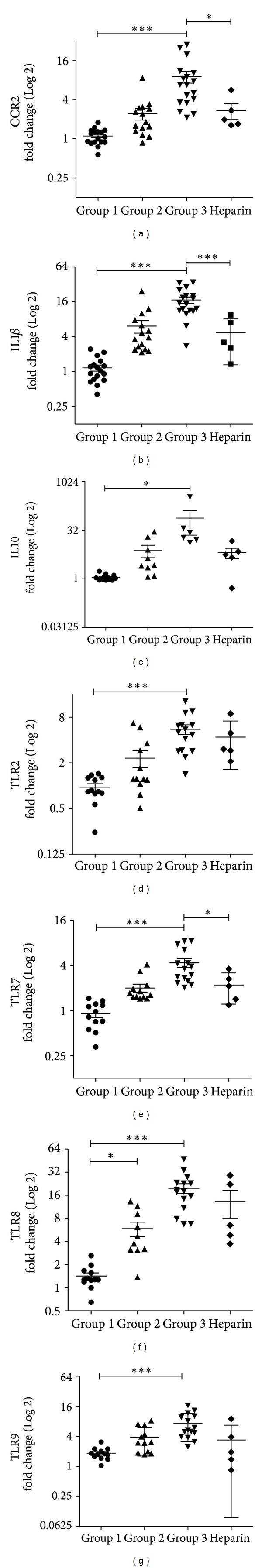
Heparin treatment affects the mRNA expression of CCR2, IL1*β*, and TLR7 in kidneys of B/W mice. The mRNA expression of CCR2 (a), IL1*β* (b), IL10 (c), TLR2 (d), and TLR7 (e) was increased in Group 3 mice compared to Group 1 mice. The mRNA expression of CCR2 (a), IL1*β* (b), and TLR7 (e) was significantly reduced in Heparin-treated mice compared to Group 3 mice. Data is given as Log 2 of mean ± SEM of fold change values normalized against 4-week-old mice (*n* = 3). *P* values are calculated using one-way ANOVA followed by Bonferroni posttest. *P < 0.05; **P < 0.01; ***P < 0.001.

**Figure 3:**
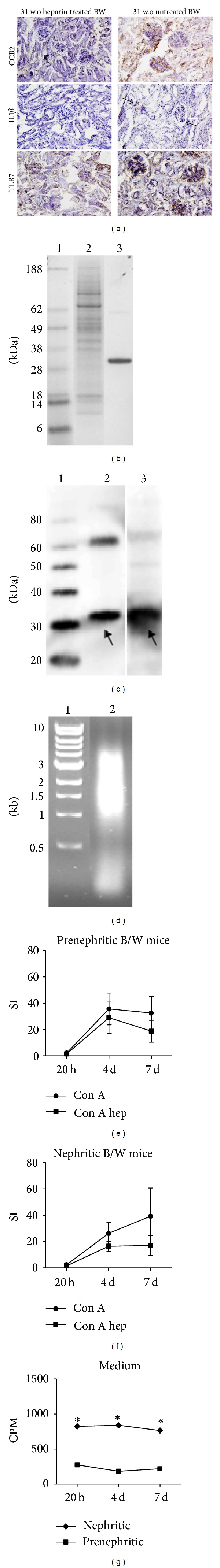
Characterization of nucleosomes used in this study and spontaneous splenocyte proliferation. Immunohistochemistry analysis detecting CCR2, IL1*β*, and TLR7 protein expression in kidneys of 31 w.o heparin or untreated B/W mice (a). SDS PAGE of nucleosomes and HMGB1 used in cell culture stimulations ((b), lane 1: MW standard, lane 2: nucleosomes (3 *μ*g measured as DNA), and lane 3: HMGB1 (1 *μ*g)). Western blot showing HMGB1 (arrows) in nucleosomes purified from A31 cell line ((c), lane 1: MW standard, lane 2: HMGB1 (0.1 *μ*g), and lane 3: nucleosomes (2 *μ*g measured as DNA)). Agarose gel electrophoresis of nucleosomes showing a distribution of nucleosome sizes ((d), lane 1: MW standard, lane 2: nucleosomes (3 *μ*g measured as DNA)). Stimulation index (SI) of cells from prenephritic mice (e) and nephritic mice (f) stimulated with conA with or without heparin. Splenocytes from nephritic mice have a significantly higher proliferation (counts per minute, CPM) at 20 hours which persisted over time compared to splenocytes from prenephritic mice (g). Mean values and SD were calculated from triplicates from each cell culture. *Statistically significant (*P* < 0.05) compared to medium stimulated cells at the same time point. Scale = 100 *μ*m.

To measure the effect of LMW heparin treatment on cytokine and chemokine expression individual TaqMan real time PCR assays (qPCR) on a selection of cytokine and chemokine genes were performed. The mRNA expression levels of CCL2, CCL7, CCL20, CXCL1, and CXCL2 were significantly upregulated in Group 3 B/W mice compared to Group 1 mice and were, although somewhat reduced, not significantly different in the heparin-treated mice (Figures 1(a)–1(f)). There were no significant increase of CCL2, CCL7, and CXCL1 mRNA expressions in age-matched BALB/c mice (Figures 1(f)–1(h)). CCR2, IL1*β*, IL10, TLR2, TLR7, TLR8, and TLR9 mRNA expressions were significantly increased in Group 3 mice (Figures 2(a)–2(g), resp.), and CCR2, IL1*β* and TLR7 mRNA expression levels were significantly lower in heparin-treated mice (Group 4) compared to nephritic mice (Group 3) (Figures 2(a), 2(b), and 2(e), resp.). Analysis of CCR2, IL1*β*, and TLR7 protein expression within the tissue verified these reduced gene expression levels observed in heparin-treated mice compared to untreated mice (Figure 3(a)). CCR2 expression was observed in tubular and glomerular areas of nephritic mice, whereas the expression in heparin-treated mice were confined to tubuli (Figure 3(a)). IL1*β* was observed in infiltrating cells that were reduced in heparin-treated mice (Figure 3(a)). TLR7 expression was observed on infiltrating cells, tubuli and glomeruli of untreated nephritic mice, and a reduced expression that was mainly observed in glomeruli and between tubuli of heparin-treated mice (Figure 3(a)). A Spearman correlation analysis (Table 1) on all parameters performed on age-matched pairs of nontreated and heparin-treated B/W mice demonstrated an inverse correlation of heparin treatment and the development of proteinuria and the gene expression of CCR2, IL1*β*, and IL10. The duration of anti-dsDNA antibody production (in weeks) correlated positively with the development of proteinuria and with expression of CCL2, CCR2, CCL20, TLR2, TLR7, CXCL1, and IL0 (Table 1). Anti-dsDNA ab production and successive deposition of immune complexes within the kidney during the disease increase the gene expression of cytokine and chemokines, while heparin treatment lowers the expression.

### 3.2. Splenocytes from Nephritic Mice Are Activated by Nucleosomes in Absence or Presence of Heparin

**Figure 4:**
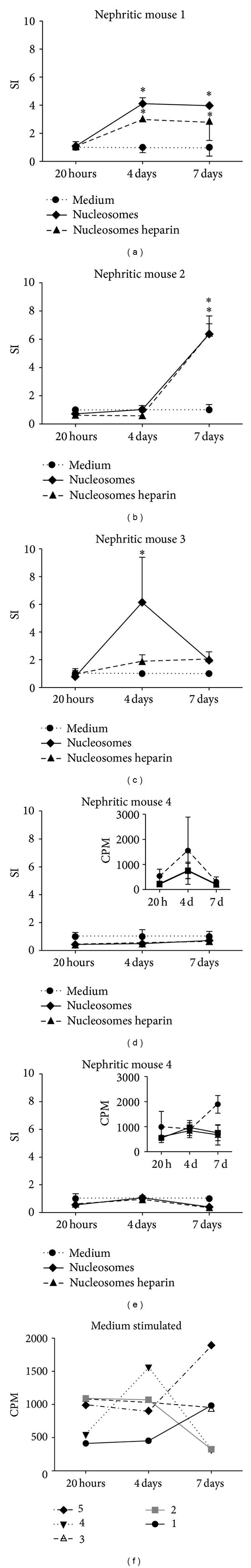
Splenocyte cell proliferation in response to nucleosomes. Splenocytes derived from three of the nephritic mice showed increased proliferation in response to nucleosomes; LMW heparin did not interfere with proliferation magnitude or kinetics ((a), (b), and (c), proliferation is shown as stimulation index (SI)). Apparently no proliferation was seen in cell cultures from two of the nephritic mice ((d), (e)), although responses in cpm indicate that the cells proliferate ((d), (e), inserted figures). Proliferations in medium for individual mice are shown as cpm (f). Mean values and SD were calculated from triplicates. *Statistically significant (*P* < 0.05) compared to medium stimulated cells at the same time point.

To analyse the effect of LMW heparin on cell proliferation, gene expression of cell activation markers, and proinflammatory cytokines, splenocytes isolated from prenephritic and nephritic B/W mice were stimulated with nucleosomes, either in absence or presence of heparin. Nucleosomes used in the present experiments contained HMGB1 (Figures 3(b) and 3(c)). The size of nucleosomes ranged from mononucleosomes to polynucleosomes (Figure 3(d)). The splenocytes from prenephritic mice did not respond to nucleosomes in any of the experiments (data not shown). However, stimulation of cells from prenephritic mice with conA resulted in a stimulation index similar to those obtained from nephritic mice with no significant reduction by heparin (Figures 3(e) and 3(f), resp.). The spontaneous proliferation in medium measured as cpm revealed that nephritic mice had a significantly higher proliferation at 20 hours which persisted over time compared to splenocytes from prenephritic mice (Figure 3(g)). Splenocytes from nephritic mice proliferated in response to nucleosomes, but the presence of LMW heparin did not affect this response (Figure 4). In three of the five mice, we observed a nucleosome-induced proliferation, while the kinetics differed between the mice (Figures 4(a)–4(c)). The presence of LMW heparin did not have any influence on the proliferation of splenocytes taken from these mice (Figures 4(a)–4(c)). Splenocytes from two nephritic mice did apparently not respond to nucleosomes (Figures 4(d) and 4(e)). Splenocytes from these mice demonstrated high initial levels of tritiated thymidine incorporation already at 20 h, indicating that they either were in a phase of proliferation when seeded into the wells, or they were fast responders giving responses before 20 h (Figures 4(d) and 4(e), (insets) and 4(f)). Stimulation with isolated HMGB1 or LMW heparin did not give a significant proliferative response in cells from any mouse tested.

**Figure 5:**
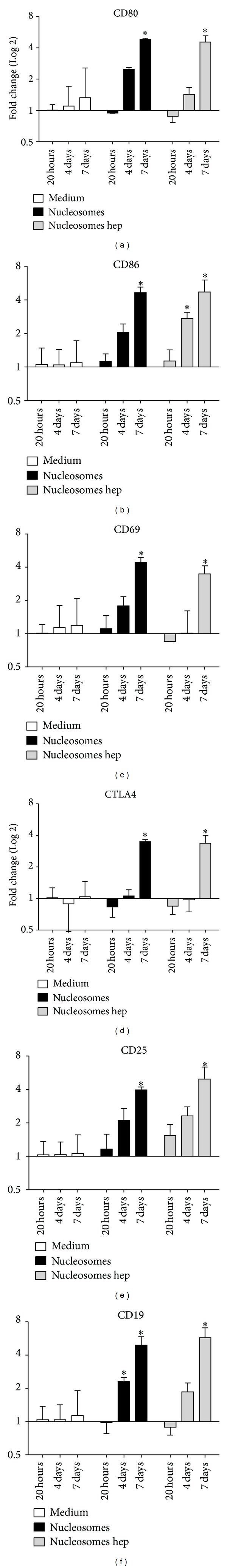
The mRNA levels of leukocyte activation markers in nucleosome- and nucleosome/heparin-stimulated splenocytes from a nephritic mouse. The mRNA levels of CD80 (a), CD86 (b), CD69 (c), CTLA-4 (d), CD25 (e), and CD19 (f) are all upregulated over time in response to stimuli with nucleosomes and nucleosomes/heparin. Expression levels for each gene, measured by qPCR, were normalized against the expression level of TBP. Data are given as Log 2 of mean ± SEM of fold change values normalized against medium stimulated cells and are representative for at least three independent analyses. Fold change is shown as mean of triplicates ± SD. *Statistically significant (*P* < 0.05) change in mRNA levels compared to medium stimulated cells at the same time point.

Nucleosome-stimulated splenocytes from nephritic B/W mice, analysed by qPCR, showed transcriptional upregulation of genes encoding cell activation markers for antigen presenting cells: CD80, CD86 (Figures 5(a) and 5(b)), activated T cells: CD69, CTLA4, and IL2 (Figures 5(c)–5(e)), and the B cell marker CD19 (Figure 5(f)). The presence of LMW heparin in the cultures did not influence the increased transcription of these markers in response to stimulation with nucleosomes (Figures 5(a)–5(f)). Responses to nucleosome stimulation with or without heparin also included an increased transcription of the genes encoding the cytokines IL1*β*, IL6, IL10, IFN-*γ*, TNF*α*, the receptors TLR2, TLR7, TLR8, TLR9, and the chemokine CXCL1 (mouse IL8 analogue) (Table 2). 

## 4. Discussion

Recent results have demonstrated that B/W mice treated with LMW heparin presented a significantly delayed and reduced anti-DNA antibody response *in vivo*. We also observed a significantly delayed development of lupus nephritis in the heparin-treated mice [19]. These results may theoretically be due to at least 2 different effects of LMW heparin. Heparin makes nucleosomes more sensitive to enzymatic degradation, and particularly to Dnase1 [19] similar to what has been described by, for example, Villeponteau [25]. This effect resulted in a nearby complete degradation of nucleosomal DNA *in vitro* [19]. LMW heparin also inhibited binding of nucleosomes to components of GBM, like laminins and collagen IV, possibly due to altered net charge and conformation of the nucleosomal structure induced by heparin [19]. These phenomenons have also been observed by van Bruggen et al. [26], although they provided a different explanation for reduced nucleosome binding to membranes.

When analysing cytokine and chemokine mRNA expression levels in the kidneys of treated and untreated mice, we demonstrated significantly reduced levels of CCR2, IL1*β*, and TLR7 in heparin-treated mice. CCR2 is mainly expressed by tubular cells in the murine kidney in addition to effector cells like macrophages. Reduced expression of CCR2 may either be because of less influx of macrophages or less expression by tubular cells. Here we also demonstrate an increased expression of CCR2 within the glomeruli of sick mice. The reduced mRNA levels of IL1*β* and TLR7 may indicate lower degree of influx of immune cells normally expressing them. Heparin has been shown to have an effect on adhesion molecules and cytokines, and can bind to chemokines [27]. In addition heparin can inhibit complement activation [28, 29]. Classical activation of the complement system also provides chemotaxis of granulocytes and macrophages through the split products of C3a-C5a [30]. LMW heparin has been shown to have an inhibitory effect on mesangial cell proliferation, signal transduction, and reduce apoptosis upon several activation stimuli [31–33]. The effect of LMW heparin on activation of mesangial cells by nucleosome and nucleosome-containing immune complexes remains to be determined. 

The ability of heparin to prevent binding of nucleosomes to membranes could theoretically also indicate that heparin could preclude binding and uptake of nucleosomes by APCs as well as binding of T-cell receptors to nucleosome-derived peptide-MHC class II complexes. This would eventually provide an explanation to the observed reduced autoimmune anti-dsDNA antibody response beyond pure degradation and loss of immunogenic nucleosomes [19]. In the present study, all nephritic mice produced antibodies to DNA, and splenocytes from 3 of them responded readily to nucleosomes *in vitro*. The reason why splenocytes from two of the five nephritic mice did not proliferate in response to nucleosomes is unclear but may be due to the fact that they seemed to be activated *in vivo* at the time they were cultured and unresponsive to further stimuli the next 7 days. This was demonstrated by high cpm values in medium-stimulated cultures already at the early phase of the cultures, and no increase in cpm was observed thereafter during the 7 days observation time. In cultures of splenocytes from Group 3 mice, T-cell activation markers together with upregulation of activation markers for APCs and B cells were observed. This was also accompanied by cytokine production reflecting a true innate immune response against nucleosomes. The cell proliferation in these cell cultures was the same when nucleosomes were presented in the presence of LMW heparin. 

In the nucleosome-stimulated splenocyte cultures derived from nephritic mice, we also observed an increase in TLR mRNA expression levels in response to nucleosomes. TLR 7 and 8 are activated by ssRNA and g-rich oligonucleotides [34, 35], while TLR 9 is activated by CpG motifs on DNA usually found on bacterial DNA [36]. They exert important roles in induction of autoimmunity [37–39]. These receptors are located in intracellular compartments of APCs, and activation of them leads to upregulation of co-stimulatory molecules and cytokine secretion needed for activation of T cells [40]. The upregulation of TLR 7, 8, and 9 seen in nucleosome-stimulated splenocytes indicates that nucleosomes activate APCs through interaction with these TLRs or that they activate cytokine production that will lead to their upregulation [41, 42]. Another TLR that has been implicated in inducing autoimmunity against nucleosomes is TLR2 which binds nuleosome-HMGB1 complexes [43]. This binding will result in activation of APCs with increased expression of costimulatory molecules required for activation of T cells [44]. The nucleosomes used in these experiments contained HMGB1 which explains the upregulation of TLR2 transcription [45]. Heparin can bind HMGB1 [46] which may dislocate HMGB1 from the nucleosomes and interfere with the binding and uptake by APCs through the TLR2 pathway. In our studies, we did not observe any difference when LMW heparin was added to the cultures. Stimulation with pure HMGB1 did not result in proliferative responses in splenocytes in agreement with previously reported results [43]. 

In this study we did not observe any inhibitory effects of LMW heparin on nucleosome-mediated activation of APCs or on proliferation of nucleosome specific T cells taken from the spleen. This indicates that even if LMW heparins bind nucleosomes and may change their net charge and conformation [19, 25, 47], it does not affect the uptake and presentation of nucleosomes to nucleosome specific T cells from mice with full-blown lupus nephritis. Thus, a more relevant explanation for the reduced anti-dsDNA antibody response in heparin-treated B/W mice would therefore be increased by enzyme-mediated elimination of the nucleosome as a central antigen [19]. Reduced load of nucleosomal antigens will lead to diminished activation of APCs, T cells, and B cells and will consequently lead to reduced amount of autoantibodies. In context of the *in vitro* experimental results described in the present study, the concentration of nucleases and proteases available in the cell cultures may be too low to affect the elimination of nucleosomes, in contrast to the concentrations observed in sera [48]. In line with this, the reduced *in vivo* mRNA levels of CCR2, IL1*β*, and TLR7 indicate that reduced levels of nucleosomal antigens might lead to reduced activation of intrinsic cells and less influx of effector cells.

## 5. Conclusion

One of the beneficial effects of LMW heparin *in vivo* in B/W mice, demonstrating delayed development of autoimmunity to nucleosomes and lupus nephritis [19], relies on its ability to lower the inflammatory processes of immune complex deposition.

## Figures and Tables

**Table 1:** Spearman correlation matrix performed on gene expression in heparin and nontreated age-matched B/W mice.

	Age	Hep.	w. ab. Pos.	Prot.	CCL2	CCR2	CCL7	CCL20	CXCL1	CXCL2	TLR2	TLR7	IL1*β*	IL10
Age		0,000	0,363	0,463	0,517	0,369	0,591	**0,640**	**0,714**	**0,739**	0,443	0,394	0,271	0,074
Heparin	1,000		−0,601	**−0,655**	−0,453	−**0,731**	−0,383	−0,522	−0,313	−0,244	−0,453	−0,592	**−0,870**	**−0,801**
Weeks antibody positive	0,303	0,066		**0,694**	**0,677**	**0,874**	0,603	**0,812**	**0,652**	0,572	**0,739**	**0,855**	0,622	**0,745**
Proteinuria	0,178	*0,040 *	*0,026 *		**0,798**	**0,798**	**0,798**	**0,798**	**0,798**	**0,798**	**0,722**	**0,722**	**0,798**	**0,722**
CCL2	0,126	0,189	*0,032 *	*0,006 *		**0,745**	**0,964**	**0,830**	0,600	**0,636**	**0,903**	**0,806**	**0,661**	**0,624**
CCR2	0,294	*0,016 *	*0,001 *	*0,006 *	*0,013 *		**0,648**	**0,891**	0,527	0,515	**0,782**	**0,879**	**0,830**	**0,915**
CCL7	0,072	0,275	0,065	*0,006 *	*<0,001 *	*0,043 *		**0,818**	**0,661**	**0,721**	**0,794**	**0,721**	0,552	0,515
CCL20	*0,046 *	0,122	*0,004 *	*0,006 *	*0,003 *	*0,001 *	*0,004 *		**0,685**	**0,733**	**0,830**	**0,915**	**0,697**	**0,770**
CXCL1	*0,020 *	0,378	*0,041 *	*0,006 *	0,067	0,117	*0,038 *	*0,029 *		**0,964**	0,576	0,576	0,479	0,333
CXCL2	*0,015 *	0,497	0,084	*0,006 *	*0,048 *	0,128	*0,019 *	*0,016 *	*<0,001 *		0,588	0,588	0,442	0,333
TLR2	0,200	0,189	*0,015 *	*0,018 *	*0,000 *	*0,008 *	*0,006 *	*0,003 *	0,082	0,074		**0,927**	**0,733**	**0,721**
TLR7	0,260	0,071	*0,002 *	*0,018 *	*0,005 *	*0,001 *	*0,019 *	*<0,001 *	0,082	0,074	*<0,001 *		**0,758**	**0,855**
IL1*β*	0,449	*0,001 *	0,055	*0,006 *	*0,038 *	*0,003 *	0,098	*0,025 *	0,162	0,200	*0,016 *	*0,011 *		**0,855**
IL10	0,839	*0,005 *	*0,013 *	*0,018 *	0,054	*<0,001 *	0,128	*0,009 *	0,347	0,347	*0,019 *	*0,002 *	*0,002 *	

**Table 2:** Transcriptional levels of cytokines and Toll-like receptors in stimulated splenocytes.

Relative gene expression levels^a^ in splenocytes from nephritic mice						
Genes	Nucleosomes			Nucleosome lmw heparin		
	20 hours	4 days	7 days	20 hours	4 days	7 days
IL1*β*	15.87 ± 5.09	77.78 ± 7.92*	72.69 ± 29.08*	15.54 ± 4.62	86.84 ± 13.09*	80.86 ± 10.80*
IL6	1.80 ± 0.42	6.91 ± 2.46	35.25 ± 8.08*	1.52 ± 0.57	6.57 ± 1.21	37.25 ± 15.09*
IL10	1.29 ± 0.53	1.90 ± 0.25	3.55 ± 0.12*	1.47 ± 0.48	1.79 ± 0.21	4.45 ± 2.16*
IFN-*γ*	2,35 ± 0.79	4,10 ± 2.62	**10,99** ± 0.38*	3,00 ± 1.18	6,53 ± 4.57	**11,20** ± 4.03*
TNF*α*	4.75 ± 0.32*	5.45 ± 0.82*	1.97 ± 0.34*	5.44 ± 1.23*	8.37 ± 1.24*	**2.04** ± 0.14*
CXCL1	110.70 ± 47.16*	51.80 ± 11.37	81.63 ± 46.31*	91.75 ± 27.24*	45.92 ± 4.40	91.75 ± 23.38*
TLR2	1.81 ± 0.75^b^	10.94 ± 0.65*	19.70 ± 4.40*	1.69 ± 0.48	10.15 ± 1.39*	19.02 ± 2.08*
TLR7	2.09 ± 0.82*	3.21 ± 0.57*	2.30 ± 0.38*	1.47 ± 0.41	2.04 ± 0.12*	2.23 ± 1.10*
TLR8	0.34 ± 0.12*	1.64 ± 0.19*	3.31 ± 0.09*	0.43 ± 0.23	1.77 ± 0.31*	2.61 ± 0.28*
TLR9	1.11 ± 0.23	2.01 ± 0.05	4.08 ± 0.87*	1.98 ± 1.05	2.76 ± 0.36*	5.10 ± 1.79*

^a^Data is given as fold change compared to medium stimulated splenocytes at the same time point. *Statistically significant (*P* < 0.05) change in mRNA levels compared to medium stimulated cells. ^b^Mean values and SD were calculated from triplicates.
